# “Play as a Nazi prison guard”: childhood and adolescent exposure to online extremist materials in online gaming environments

**DOI:** 10.3389/fpsyg.2025.1504584

**Published:** 2026-02-06

**Authors:** Jade Hutchinson, Ruxandra Mihaela Gheorghe, David Yuzva Clement, Kenton Bell, Lorraine Kellum, Michaela Rana, Alex Shuttleworth, Stephanie Scott-Smith

**Affiliations:** 1 Department of Security Studies, Post-Doctoral Fellow in the Faculty of Social Sciences, Charles University, Staré Město, Czechia; 2 School of Social Work, Carleton University, Ottawa, ON, Canada; 3 Independent Researcher, Mooresville, NC, United States; 4 Educational Consultant, Teachstone Inc. M.A. in Education, Virginia Tech, Blacksburg, VA, United States; 5 Department of Security Studies and Criminology, Macquarie University, Sydney, NSW, Australia; 6 The School of Psychology, University of New South Wales, Sydney, NSW, Australia; 7 Department of Security Studies and Criminology, Macquarie University, Sydney, NSW, Australia

**Keywords:** online gaming, children, adolescent, extremism, radicalization, video games, development

## Abstract

This article analyzes 18 expert assessments reflecting professional evaluations and experiences on the relationship between children, adolescents, and online gaming technologies that facilitate exposure to extremist contents and recruiters. We conducted semi-structured interviews with 18 researchers, practitioners, and policymakers from government, academic, and education-based organizations across the Indo-Pacific, Europe, and North America. Online gaming emerged as a prominent concern and important component of the sociotechnical environment that facilitates children and adolescent exposure to extremist content and recruitment activities. The findings emphasized the importance of private–public partnerships, future “safety-by-design” initiatives, interdisciplinary collaboration with the cognitive-psychological and the developmental sciences, and need to understand the swiftly changing technological characteristics of online gaming in shaping how children and adolescents may encounter online extremism. We also highlight these experts’ opinions on the wider sociotechnical environment where such exposure is made possible. This article offers guidance and recommendations to researchers, practitioners, and policymakers who wish to better understand and address the influence of online extremism on child and adolescent development in the digital age.

## Introduction

Children and adolescents spend a significant amount of time on online gaming platforms and within their associated communities (Meltwater and We Are Social, 2024).^1^ These spaces are considered opaque online environments that are difficult to monitor or regulate, where younger and less-mature users navigate encrypted gaming chat rooms (Yuzva Clement et al., 2023). Extremist actors and recruiters leverage design and operational features of online gaming technologies—and bordering social networking platforms and platform designs—to converse with and convince younger and more vulnerable users of their ideologies and communities (Koehler et al., 2022; Koehler et al., 2023). Security and intelligence agencies from Australia, Canada, New Zealand, the United Kingdom, and United States have recently warned the public about the presence of “ideologically motivated violent extremists” in online gaming-adjacent environments, where recruiters are “targeting youth online with the intention of getting them to record or live-stream acts of self-harm, suicide, animal torture and/or produce child sexual abuse material” (see Royal Canadian Mounted Police, 2024). Younger users within large online multiplayer games like Minecraft and Roblox are particularly susceptible to extreme cultural authorities or influencer personalities without sufficient supervision or safety-by-design features such as age-appropriate restrictions (Vittozzi, 2020; Hartgers and Leidig, 2023; Koehler et al., 2022; Koehler et al., 2023). While our focus is on platforms frequently accessed by younger demographics, such as Minecraft and Roblox, the broader implications extend to all multiplayer gaming environments. For instance, concerns about “online youth radicalization” continue to prompt investigations into various factors that facilitate a child or adolescent’s exposure to, encounters with, and experiences with extremism in various online gaming environments (Kowert et al., 2024; Schlegel and Kowert, 2024). Understanding these operational features addresses certain current vulnerabilities that policies and programs must address.

Policies and programming aimed at curbing online extremism among young people encompasses a range of counter radicalization interventions, including—but not limited to—content moderation practices, civic education, capacity building trainings, training of trainers, digital media literacy, disengagement interventions, family-based interventions, and involving younger audiences in shaping their nation’s online safety governance policies. But particular uncertainties persist (Yuzva Clement et al., 2023; Hutchinson et al., 2025). There are developmentally relevant factors that are subsumed in most assessments of “online youth radicalization,” resulting in unresolved and unexplored intersections that are critical to understanding and addressing this issue. For instance, there are questions revolving whether and to what degree the concept of “radicalization” accurately encapsulates or represents the social and cognitive-psychological realities of children and adolescents when exposed to extremist materials or contacts (see Campelo et al., 2022; Hutchinson et al., 2025). Additionally, there are practically relevant factors that remain important yet unaddressed and untested, including the accountability of reporting in different countries which results in considerations of whether comprehensive “whole-of-society” approaches or private–public partnerships or short-term funding cycles, are effective and plausible strategic pathways for mitigating the harms of—what has been recently labeled—“online gaming ecosystems” (Lamphere-Englund and White, 2023; Yuzva Clement et al., 2023; Moonshot, 2024).

## Methods

### Research focus

In this context, researchers, practitioners, and policymakers are searching for reflections and answers about where exposure takes place and when children and adolescents are particularly developmentally sensitive to certain digital media environments or ideological influence and why. This article examines the insights and recommendations of 18 experts from academic, private, and governmental institutions to identify the key areas for future research and policy development aimed at enhancing the understanding and mitigating “online youth radicalization.” Our objective was to capture valuable professional assessments, contextual insights, and recommendations from experts concerning the relationship between children and adolescents and their exposure to extremism in online settings. Throughout our expert interviews, online gaming emerged as one of the prominent element of the sociotechnical landscape that was said to effectively facilitate the child or adolescent’s engagement with ideological content or communications. The central argument of this research is that while various policies and practices exist (and continue to arise) to address online radicalization with children and adolescents, they remain fragmented, reactive, short-term, and insufficiently attuned to the unique social and technological contexts, as well as neurodevelopmental vulnerabilities of younger populations. Without a comprehensive engagement with these realities and proactive, long-term policy-investment initiatives, stakeholders—including children, adolescents and their families—will face challenges in responding to the rapidly evolving nature of online youth radicalization.

To address this issue, the study is guided by the following overarching research question:

“*What policies and practices are currently in place to tackle the challenge of radicalized minors?”* This question is further divided into three key sub-questions:

- What policies and practices are currently being considered, proposed, or employed to understand and mitigate the harm of (online) extremism on children and adolescents?
- What are the implications or possible consequences of the current policies and practices (assuming the current trajectory)?
- Considering this, what are recommended policies or practices that are likely to improve efforts to mitigate potential harms caused by (online) extremism on children and adolescents?

### Semi-structured interviews

#### Sampling and recruitment

The 18 semi-structured interviews were conducted between July 2023 and July 2024. Interviewees were identified—either by the authors or through engagement with the European Union’s former *Radicalization Awareness Network*—as experts in child or adolescent radicalization, policies and programming concerning preventing or countering radicalization, violent extremism among vulnerable online populations of young people, expertise in online extremism generally, or engagement in youth-oriented prevention and countering violent extremism (P/CVE) programs. Participants were provided informed consent prior to their involvement in the study, ensuring they fully understood the nature, objectives, and potential implications of their participation. They were explicitly informed about their right to withdraw from the study at any time without consequence. Participants were also briefed on the measures taken to safeguard their interviewee data, including confidentiality and data management protocols, with minimal demographic data pertaining to each participant recorded for displaying research results.

We interviewed experts and stakeholders from diverse occupational, geographical, and institutional backgrounds. The 18 participants were drawn from 14 countries, evenly split by gender, and represented a wide range of institutions and areas of expertise, including law enforcement, policy, academia, education, and mental health industries (see Table 1).

**Table 1:** details the unidentifiable demographic information and corresponding pseudonyms of the 18 interviewees.

Pseudonym	Sector	Organization	Gender identified	Country of operation
Expert 01	Academic/research	Think tank/professional organization	Female	United Kingdom
Expert 02	Practice	Private company	Male	Canada/United States
Expert 03	Academic/research	University	Male	Australia
Expert 04	Academic/research	Research institute	Female	Germany
Expert 05	Practice	Non-profit organization	Female	Canada
Expert 06	Researcher/practice	Non-profit organization	Male	Canada
Expert 07	Academic/researcher	Professional organization	Female	Bulgaria
Expert 08	Practice/policy	Government agency	Male	The Netherlands
Expert 09	Policy/researcher	Government agency	Male	Belgium
Expert 10	Academic/researcher	Research institute	Female	Germany
Expert 11	Policy	Government agency	Female	Germany
Expert 12	Academic/Policy	Non-profit organization	Male	Sweden
Expert 13	Policy maker/policy advisor	Government agency	Male	Denmark
Expert 14	Academic/researcher/practice	Professional organization	Male	Austria
Expert 15	Practice/policy advisor	Professional organization	Female	Romania
Expert 16	Academic/researcher/policy advisor	Inter-government agency	Female	Italy/France
Expert 17	Policy maker/policy advisor/practice	Government agency	Female	United Kingdom
Expert 18	Academic/researcher	University	Male	United Kingdom

Finally, the research team’s positionality and power dynamics were carefully considered to uphold ethical engagement with participants. As interviewees were professionals with pre-existing stakes in research, policy, or intervention frameworks, efforts were made to minimize any perceived hierarchy or influence during interviews. Particular attention was given to identifying areas of agreement and contention among interviewees—especially regarding policy regulatory responsibility, the commercial role of gaming companies, and the balance between child protections and digital autonomy—during sampling and recruitment stages.

#### Data collection

The interviews were semi-structured to capture expert perspectives and assessments on how and why children and adolescents engage with online extremist materials within digital media environments, such as online gaming platforms, and to examine how policies and programming are directed at “online youth radicalization” currently and in the future. This interview method offered participants the flexibility and freedom to share their own perspectives, experiences, and assessments without feeling constricted by a predetermined set of structured questions. Interviews were guided by prepared questions to maintain consistency, such as:

- Why and how do extremist actors target children through online propaganda?
- Where, who, and when do children or adolescents encounter extremist content online?
- What are the effects or consequences of exposure on childhood or adolescent development?
- What are the lessons and limitations of current approaches and policies to “online youth radicalization”?
- How can these lessons and limitations help researchers and policymakers to develop measures and preventative strategies to better understand and counter child and adolescent exposure to harmful extremist content online?

Interviews were conducted via the video web conference site *Zoom* and lasted between 45 and 90 min. All interviews were anonymized and transcribed verbatim using the language processing software NVivo. All collected data were stored in encrypted files on secure servers, accessible only to the research team. This study focused on children and adolescents aged approximately 7–14, recognizing significant individual variation in developmental maturity (Yuzva Clement et al., 2023). Subsequently, we asked interviewees to—where possible—differentiate between different developmental phases (e.g., children, or early or late adolescents) when providing their assessments and reflections. This study design was reviewed and approved by the International Centre for Counter-Terrorism in collaboration with the former European Union’s Radicalization Awareness Network. Ethical procedures included informed consent, anonymization, secure data handling, and clear communication about the study’s aims, use of findings, and potential risks.

#### Data analysis

Transcripts were analyzed using a six-step reflexive, thematic analysis approach, where the theme codes were initially generated by members of the research team to categorize overarching narratives and themes that had emerged in each interview, establishing commonalities and differences between expert assessments and reflections (Braun and Clarke, 2021). The findings of this thematic analysis were also iteratively refined through further assessments. Following data collection and the initial thematic analysis, each transcript was re-examined in NVivo to statistically determine the proportionate use of meta-themes and associations across the interviewees’ viewpoints (McEnery and Hardie, 2011; Brezina, 2018). Each author individually conducted a secondary thematic assessment to enhance intercoder reliability and comparison, ensuring that the identified themes were not only subjectively derived from researcher assessments of experts’ discourse, but significant to the broader dataset and contextualized in conversation.

## Results

Findings are presented across three themes emerging from the interviews: (1) overlooked and unknown; (2) developmental sciences and behaviors; and (3) exposure to extreme materials.

### Overlooked and unknown

Most interviewees agreed that because digital media environments evolve so quickly, so do youth experiences of extremism online. Interviewees expressed concerned about communities and platforms associated with online gaming—such as mass-multiplayer sandbox games or role-playing games including Call of Duty, Minecraft, and Roblox—social network platforms including TikTok, X, Snapchat, and Meta, and streaming or video chat services, including YouTube, Twitch, or Discord, which are known to host recruitment of and/or are suspected to harbor ideologically extreme content. Rather than the “content,” these interviewees’ concerns encompassed widespread usage and accessibility to “alternative,” “semi-public,” and “underground” online gaming adjacent “communities” and that can propagate “toxicity,” “extremist ideas,” “hate speech,” and “violent extremism” online. Interviewees suggested that online games can be “really positive social engagement” exercises, while others mentioned the known risks, including “grooming” and the “very vast space” that composes online gaming communities, making it challenging for counter violent extremism efforts to monitor. As one interviewee stated:

“It’s not on the immediate surface, but pretty much as soon as you start scratching below that surface, you are going to find [it] if you go for it. So, while these games, while these spaces, gaming adjacent spaces are pitched as being for children and they were designed to be for younger age groups, they still harbor this type of ‘people content’ and they are called sandbox games. But that’s basically where, you know, a user can go out and build their own environments in that game. So Roblox is a good example because it’s not that Roblox is putting Nazi prison guard content out there. It’s that a user of Roblox is able to go into the Roblox game and build that environment themselves.” (Expert 01)

However, in every interview was the acknowledgement that children and adolescents are “overlooked” or “underrepresented” or “ignored” in counter violent extremism programming targeting online extremism in general and in online gaming especially. Interviewees provided various reasons for why these oversights may exist. For example, several practitioners emphasized that there are few practical or legislative instruments “specifically on youth and children” available to those working in counterterrorism or counter violent extremism. As one interview, stated:

“There are the real investigations of cases where the child is often forgotten because they would be an informant or a witness or a victim. But this site is often forgotten because then the focus is on tracing the activities of the terrorists.” (Expert 16)

Most interviewees strongly cautioned against radicalization frameworks as they can unnecessarily combine assumptions about adult radicalization with assumptions on the development of children and adolescents. As one interviewee described:

“We either look at them as adults, and therefore, it’s justified to treat them as adults and they need to be punished, or they are only victims. And therefore, we completely overlook their agency. And I think we need to understand children are both—they are not the same developmental level as an adult, so they cannot be ascribed the same responsibilities, but they are not stupid, so they can be part of the process… And this is something that I think terrorist tactics have understood a lot better than governments. So not to look at children as if they are inherently less apt at making choices than adults are. So, they really value children. And I think this is what the children are responding to in these recruitment processes.” (Expert 16)

For instance, one interviewee emphasized how sensitivity to radicalization frameworks can influence how practitioners approached a counter violent extremism case involving children:

“I’ve seen… situations where children have been reported to go through the [governmental] mechanisms for potential extremist views, where actually, there was nothing to do with extremist views. That creates tension, mistrust, and it actually creates outcry sometimes…I can think of one very infamous situation in the UK where it was alleged a four-year-old had been reported to [governmental programs] by a nursery on the basis of something he’d drawn. And the interpretation of this drawing was that it was somehow linked to violent things that he was seeing at home, something terrorist in nature. And actually, it turned out that this child and when he was asked what it was, he was mispronouncing what it was, and it sounded like he was referencing some kind of ‘bomb’, but actually, he was trying to say the word cucumber and it was completely misunderstood.” (Expert 17)

Subsequently, another interviewee suggested that using these frames attracts national government funding without mitigating for over-securitizing youth populations in counterterrorism, stating:

“Terrorism is considered a national security threat and anything that’s considered a national security threat is going to be out funded and out discussed more than any other harm, no matter how significant it is…I think there might be benefits of rolling issues of extremism into this broader digital education and online harms. But at the same time, it will always end up dominating. It will dominate in terms of funding, and it will dominate in terms of government.” (Expert 03)

Although our findings reveal experts’ reservations toward traditional radicalization frameworks, our analysis critically engages these frameworks to highlight their limitations and propose developmental alternatives.

### Developmental sciences and behaviors

The results of the interviews overwhelmingly highlighted the scant interdisciplinary collaboration between extremism and terrorism studies and the developmental or cognitive-psychological sciences. The developmental cognitive psychology of children and adolescents was considered to be foundational to understanding their vulnerability to online extremism generally and online radicalization within online gaming communities. For instance, interviewees referenced “evolutionary perspectives” of the “delayed maturation of the teenage brain.” With one interviewee highlighting that distinct stages in brain development supposedly correspond with different levels of vulnerability, with teenagers being “extra vulnerable” and less “mature” than “after age 26, when the brain has matured.” Most interviewees mentioned deficit-focused perspectives, referencing how teenagers express a constrained capacity for “rational thinking,” “empathy,” “prudence,” “risk awareness,” and “emotional regulation,” leading to “problematic,” “risky,” “impetuous,” “troublesome,” “vulnerable,” as well as “gullible” behaviors such as “play” that are partnered with a lack of “skills,” “competence,” and “self-reflection” while online. As an interviewee stated:

“When you are 7–14, but up until 18, you are developing, you are a child… I think children are excited by things…the child’s kind of mentality of being excited by different things and unusual things and things that appear to be fantastic. An impact will get drawn down because that’s a child’s curiosity or person’s curiosity. So, I think that can be a vulnerability.” (Expert 17)

This is coupled with interviewees at times describing these digital media technologies as being more than “a tool,” framing it as an “enabling environment” integral to the personal development of children and adolescents simply by “going online these days.” Interviews highlighted the various “modes and nodes” through which young individuals interact with technology, with interviewees emphasizing the pervasive presence of “gaming devices,” “mobile devices,” and other “digital devices” in households, leading to extended screen time and increased exposure to online extremism.

Interviewees also frequently referenced hypothetical descriptions of “online youth radicalization” involving “neurodivergent” children or adolescents present in online gaming adjacent communities. Neurodivergence refers to the variation in neurological development and cognitive functioning, including conditions such as autism, attention-deficit hyperactivity disorder, and dyslexia among many others. Neurodivergent people generally process information, communicate, and interact with their environment differently from neurotypical norms. Acknowledging neurodivergent traits allows for tailored interventions within existing digital literacy and online safety programs. For instance, some participants had suggested an “autistic” child or adolescent may be more susceptible to extremist recruiters and mass-grooming strategies because they may experience online gaming as emotionally unintrusive and, therefore, may be more likely to interpret recruitment attempts with less suspicion. As one interviewee stated:

“Over the last years, we have seen more and more crossover between radicalization processes and psychiatry, for example, radicalization processes and autism. They need black-and-white things to be in balance. They need to be with somebody. But they are challenged to be with somebody physically. But it’s much easier for them to be…online with somebody playing with them…and being very, very, very good at gaming.” (Expert 13)

Adult academics and practitioners described “developmental behaviors” differently, signaling to a possible misunderstanding rooted in adult perceptions of what development is and what its associated behaviors “looks like” during childhood and adolescence. Although every participant offered one or multiple explanatory neuro-developmental qualities underlying youth vulnerability to radicalization, a core interdisciplinary “gap” often emerged and punctuated their reflections. As one interviewee stated:

“I think children…are still in the process of forming their ideas about the world…So if you allow these influences in at that young age when they are still forming their beliefs, then you are inviting that to be become part of their beliefs…I think [children] would have less tools at their disposal mentally and emotionally to think through that all the way, which I assume was probably what makes it more dangerous for kids. But I’m not a child psychology expert, so I’m not qualified really to answer that question.” (Expert 01)

Developmental preconditions were considered an increasingly significant component of age-appropriate counter violent extremism programming. Education policy or institutional practices were frequently mentioned in discussions, including the “role of parents and curriculum” in cultivating “critical thinking” or “self-reflection” skills, like media and information literacy or mindfulness meditation practice. For example, one interviewee recommended teaching children and adolescents how online gaming platforms and adjacent communities are highly emotional places, where their psychological well-being (i.e., “attitude” and “mood”) and opinion and beliefs (i.e., “ideology”) are subject to considerable influences.

Interviewees who discussed the emotional and cognitive effects of online gaming adjacent communities, terms such as “competitive,” “combative,” “arousing,” and “hyper-stimulating” frequently appeared as symptoms that self-regulatory practices can help to mitigate. However, practices such as meditation or mindfulness may not—as some interviewees suggested—sufficiently equip children and adolescents with the necessary tools to safely engage challenging subjects or sentiments that are otherwise absent from everyday life, like extreme beliefs, victimhood narratives, grooming strategies, or staunch expressions of hatred. While cognitive-behavioral techniques were proposed as suitable interventions for countering “online youth radicalization” in online gaming and online gaming adjacent communities, exactly how to design or tailor self-regulatory practices for different developmental stages remained unclear. Although interviewees frequently referenced psychology to understand children’s minds in the context of online radicalization, they acknowledged that links between developmental milestones and radicalization remain vague. Despite its importance, developmental science is underutilized in current CVE policy, creating a critical gap. Addressing this gap may enable more targeted interventions that align with young people’s different stages of cognitive and emotional development.

### Exposure to extreme materials

Several interviewees stressed the need to understand the threat of misogynistic and manospheric content as a “soft entry point” to extremism in gaming communities. For instance, extreme misogynistic materials in popular social media or online gaming platforms were characterized as “attractive” or “striking” to—especially male—children or teenagers with propensities, interests, frustrations, insecurities, ignorance, or any additional anti-social grievance that increases their susceptibility to misogynistic viewpoints. Interviewees noted misogynistic content—stemming from “manospheric movements” adjacent to online gaming communities—is circulated in gaming chatrooms and was made familiar through “influencers” in adjacent networking platforms, speculating that an incremental introduction to misogynistic or other ideologically radical content evoked “positive associations” between individuals—as young as “six-year-old”—and the influencer whom the child is exposed. Interviewees had frequently described the psychological appeal of influencers with extreme misogynistic content, with the name “Andrew Tate” appearing in almost every interview conversation alongside descriptions of their content such as “desirability,” “suitability,” or “attractiveness.”^2^

Interviewees highlighted how extremist content can be tailored to online games using popularized subcultural symbols and ancillary social network technologies to further persuasiveness. For instance, one participant suggested that online gaming infrastructure is “fundamental” to emotional and social relationships between youths and extremist content or actors because it offers plenty opportunity for trusted social cooperation in pursuing fantastical in-game “goals” like “slay the dragon,” which—according to our interviewees—united “real and cyber realities” during recruitment. However, some interviewees emphasized that whatever presumed effectiveness subcultural viral misogyny, or the design of software architectures has on children or an adolescent, depends (in part) on their unregulated yearning to “fit in with their peers” or “belong” and whether they previously experienced an “adversity” or “trauma.” As one interviewee stated:

“There’s a lot of trauma. So, we had a quite a push on trauma informed practice for those children and wrote that into the work that we wanted practitioners to complete…So, any kind of child with adversity, parental neglect, children’s own needs, children’s own vulnerabilities around their own mental health, their own ability to understand in a wider political and kind of population kind of understanding what’s going on around them and their understanding of their identity… Because they have an initial mental health problem that connects with…isolation and that generates the need for such a child to find some companionship at least online.” (Expert 17)

These adversities were considered particularly influential in connecting the child or the adolescent to gaming communities where extremist materials and contacts are present. For example, one interviewee described this with reference to their practitioner experiences managing younger clients where online gaming was involved:

“[Children and adolescents can be] dissatisfied or discontent with something [or] being bullied [or] build up some hate for something [or experiencing] mistreatment at home [or] being subject to violence or just ostracized [in ways so you are] much more easily drawn into these kind of groups…People who are having trouble adapting socially in real life get more easily get drawn into gaming…I mean gaming full-time…you know, sitting people who are we have what is called …a ‘sitter’. It’s a switch for people who do not go to school. You know, it’s impossible to get them to go to school. And they stay at home playing video games, doing things on the Internet all the time. But it’s a growing population, really. And I think that’s the case all over the world. And because it’s partly because it’s easier to interact socially online. There’s a vulnerability.” (Expert 12)

Aside from psychological or social traumas, the interviewees frequently referenced circumstances that extended beyond individual differences. For example, the COVID-19 pandemic was considered a “clear historical moment” when children and adolescents were disconnected from various mitigating factors and their own vulnerability to extremism had increased dramatically. As one interviewee suggested:

“I think COVID had a massive impact, you know, children isolated from other narratives…During the COVID period, there was a need to find a way to deal with children at home…who were very vulnerable to being influenced through different mechanisms…they do not talk in school, they talk online, especially with the pandemic, it became so normalized to have Zoom lessons, Zoom discussions, WhatsApp groups with parents, WhatsApp subgroups, with the educators WhatsApp group between them. So, everything is online now…young people who do not have a variety of counter narratives around them only have access to one viewpoint, and then that’s manipulated… [children and adolescents] need to find some resources somewhere and they find it online, and they become radicalized [and engage with] extremism online because they are depressed, they are anxious, they are isolated, [and] they have trauma. They have some other problems. And it is all interconnected.” (Expert 17)

Interestingly, despite the focus on this technological immersion and what the “structural” measures are to prevent “harm,” “abuse,” “exploitation,” “grooming,” “crime,” or other “threats,” interviewees did not frequently reference technical practices or policies that are available to technology companies who manage online gaming platforms, including more severe “deplatforming” or “content moderation.”

## Discussion

The following sections discuss the important practical implications for existing counter radicalization and counter violent extremism approaches to childhood and adolescent exposure to extremist materials in online gaming environments, while pointing to the future opportunities for programming and avenues for research.

### Policy implications

Country-level policies typically require children to be supervised by licensed childcare workers. However, findings suggest that youths identified as “victims” may face challenges when childcare providers struggle to recognize their developmental needs and instead place them in rehabilitation spaces; meanwhile, youths identified as “potential offenders” may face additional challenges stemming from the criminal justice system’s impact on their socio-emotional development. For instance, these findings revealed an important contradiction: Children and adolescents are often disproportionately targeted as being at risk of radicalization, yet their developmental needs are frequently overlooked in deradicalization contexts. Interviewees frequently recommended a multifactorial approach—predominately drawing from public health models or interdisciplinary collaboration related to the developmental sciences—as traditional counterterrorism methods are likely to be insufficient in addressing online populations of vulnerable youth (Bronsard et al., 2022). Implementing these approaches with a better understanding of child and adolescent neurodevelopment in the context of online gaming platforms, and their adjacent communities, can enhance the immediate circumstances of vulnerable young gamers potentially exposed to online extremism (Reer et al., 2024). Video games’ cultural and emotional appeals can be harnessed by further collaborating with developers or validated players in mentorship programs for online multiplayer games to foster positive peer networks as a resilience strategy against extremist messaging rewarding the participant and “mentor” with in-game “rewards,” for example.

A critical challenge in countering youth radicalization in online gaming platforms is the increased difficulty in disentangling them from the surrounding social media platforms. Contemporary “online gaming ecosystems” extend well beyond the games themselves, encompassing a network of interconnected semi-public and access-restricted platforms which increase the number of possible vectors for online extremism to circulate. Online gaming adjacent platforms, such as Discord, Twitch, and Reddit, have further blurred the boundaries between online gaming and social media platforms, creating hybrid digital media environments where extreme content can circulate with minimal capability for monitoring or parental oversight. Gaming adjacent platforms are designed to facilitate real-time communication, community-building, and user-generated content, yet they remain largely outside the scope of traditional content moderation frameworks used by online gaming companies. This fluidity has complicated regulatory efforts, which have historically defined online gaming and social networking platforms as distinct online domains. While policies such as Australia’s Online Safety Act (2021), the European Union’s Digital Services Act (2022), and the UK’s Online Safety Act (2023) establish obligations for digital media platforms to detect and remove harmful content, their applicability to a dynamic and interactive global network of gaming and gaming adjacent platforms where each platform has varying policies on content moderation remains ambiguous (see European Union, 2023; eSafety Commissioner, 2024; FTI Consulting, 2024). This structure, or lack of thereof, allows harmful content—including extremist ideologies, hate speech, recruitment, and radicalization—to traverse platforms, proliferate, and fragment in ways that can evade detection. For example, Twitch has faced criticism for failing to prevent radicalizing materials in chatrooms and live discussions, where concerns have been raised about the amplification of antisemitism on the platform, with specific streamers accused of spreading hate speech and endorsing violence against Jewish people (Christenson, 2024).

These complexities point to several priority areas for future research and policy development concerning gaming companies and civil society. First, research is needed to examine how regulatory frameworks can meaningfully incorporate gaming-adjacent platforms into broader online safety initiatives internationally. This includes tracing the circulation of content and interactions across interconnected platforms and identifying the functional overlaps between online gaming and social media environments. As online gaming platforms exert increasing influence over the everyday lives of children and adolescents, particularly those navigating vulnerability, exclusion, or trauma, regulatory mechanisms must be equipped to respond with the same level of scrutiny applied to social networking technologies currently channeling online gaming sites.

Second, future studies should investigate the mechanisms and conditions under which gaming companies can meaningfully collaborate with policymakers. This involves examining models of co-regulation, shared accountability, and the translation of child protection principles into the design and governance of multiplayer ecosystems and their adjacent communities. Third, there is a growing need to evaluate the capabilities and limitations of automated content moderation tools in gaming environments. Unlike traditional social media platforms, real-time interactions and ephemeral content in gaming spaces present unique technical and ethical challenges for detection.

Finally, further inquiry is required to design interventions that safeguard vulnerable users while preserving the participatory norms and creative freedoms integral to gaming cultures. Research should explore how protective measures, such as in-game warnings, adaptive community guidelines, or age-specific moderation layers, can be embedded without undermining user autonomy or fueling resistance among gaming subcultures. As online gaming platforms increasingly shape identity development, social belonging, and meaning-making for young communities, it becomes essential to understand how design, engagement, and emotional resonance can be leveraged for protective purposes. This work is especially important in light of the growing presence of gaming platforms in digitally mediated peer cultures, where the lines between play, expression, and ideological exposure are increasingly blurred.

Furthermore, our findings emphasized that different bodies have a critical role to play in ensuring effective and tailored localized interventions, including policymakers, but also child and youth serving organizations. These partnerships could efficiently facilitate the sharing of knowledge and resources, enabling the gaming industry to align its efforts with those groups connected to the effects of their technological products. However, as private corporations are often reluctant to act without market incentives or government mandates, there is a pressing need for regulatory frameworks that explicitly addresses the intersection of online gaming, adjoining social media platforms, and online extremist communities. These frameworks can be designed to accommodate for challenges posed by gaming environments, where real-time interaction, anonymity, and user-generated content are characteristic of user participation (assuming age limitations are applied), and in this context, regulatory incentives can also encourage greater industry participation. While government mandates have provided a strong foundation—the European Union’s Digital Services Act (2022), for example—they must be first complemented by public awareness campaigns and industry-driven accountability in their design, which together can create a culture of responsibility within the gaming industry (Weil, 2023).

Public-private research into the online gaming industry may produce opportunities to better leverage in-game software designs and insights from child developmental science or organizations that promote prosocial behaviors among young players. The cultural and emotional appeal of online gaming can be harnessed by collaborating with developers or validated players in mentorship programs for online multiplayer games to foster positive peer networks and resilience against extremist messaging. For instance, practical peer-support interventions could include in-game mentorship schemes where older or vetted players help younger users navigate risky interactions, rewarding the participants and the “mentor” with in-game “rewards.” Multiple interviewees envisioned future educational programs that encouraged online gamers to think critically and be more digitally literate, as well as incorporate cognitive behavioral techniques into online safety measures. However, the effectiveness of placing online safeguards in gaming software architecture may be far more likely when thwarting extreme online material, rather than assuming children and adolescents will adopt—accurately and consistently—practices of introspection and self-awareness beyond their stage of development. Accordingly, “safety by design” approaches are increasingly recognized as essential to mitigate the risks associated with children’s use of augmented and virtual reality games, such as the United Kingdom’s “Age-Appropriate Design Code” (Children’s Code) which requires platforms popular with children—like Roblox and Minecraft—to incorporate features that include age verification, limited data collection, and default privacy settings aimed at ensuring safety (Information Commissioner’s Office, 2023, 2024).

### Internal change and maturity

Overall findings emphasized the need for age-appropriate and multifaceted intervention measures rather than simply adapting adult-focused P/CVE strategies. P/CVE professionals whose work impacts children and adolescents would benefit from training in child development, neurodivergence, and the impact of trauma to enhance their understanding of the unique risks, needs and vulnerabilities that can present in this cohort. Greater multidisciplinary collaboration, including with experts from cognitive-psychological and developmental sciences may be needed to achieve this. Interviewees stressed that childhood and adolescence are two decisive stages of cultural, social, and emotional development. However, the underlying neurodevelopmental mechanisms that affect these stages were less frequently discussed. A critical neurodevelopmental factor is delayed frontal cortical maturation. The human prefrontal cortex contributes to executive functions such as decision-making, impulse control, and abstract reasoning (Sapolsky, 2017; Bronsard et al., 2022; Campelo et al., 2022). These capacities do not fully develop until around 24–26 years of age (Casey et al., 2008). Prior to this maturation, children and adolescents are especially susceptible to social pressure, cultural cues, and reward signals (Sapolsky, 2017). Neurodevelopmental immaturity heightens the sensitivity and malleability of young individuals to external influences (Steinberg, 2008). Such developmental vulnerabilities are exacerbated by the immersive and emotionally charged environments of online gaming platforms. These platforms often foster peer pressure and the need for social acceptance, which can increase exposure to extremist ideologies and manipulation (Crone et al., 2016). Experts noted that younger teens are especially vulnerable to influential online personalities and graphic content, struggling to differentiate between gaming expertise and credibility in other areas (Anderson and Warburton, 2012). Adolescents may inadvertently spread extremist content through their participation in online gaming cultures, blending extremist ideologies with subcultural expressions (ICSR Team, 2021). Our interview results reveal that experts were concerned with “cognitive development,” “maturation” and “emotion” however also enquired how neurological milestones relate to radicalization pathways especially when online. This article underscores the importance of studying the unique developmental trends and needs of children and adolescents, distinct from adult radicalization models. Understanding how neurodevelopmental factors intersect with online environments is crucial for developing effective prevention and intervention strategies in the context of online gaming and extremism.

### Gaming architecture and adverse experiences

Our findings identified the specific features of online gaming platforms that facilitate children’s and adolescents’ engagement with extremist content. Interviewees pointed to how the architecture of these platforms—including customizable avatars, closed chatrooms, instant notifications, anonymity, and algorithm-driven content—creates environments ripe for manipulation and radicalization. For instance, extreme misogynistic content and influencers from the manosphere have become accessible and familiar to younger users through online gaming. Interviewees noted that figures such as Andrew Tate have influenced children as young as six by embedding their personas into gaming environments. In games like Minecraft, users can customize their characters with “skins” that reflect manospheric symbolism, subtly normalizing harmful ideologies (Planet Minecraft Community, 2024). This exposure creates positive associations with controversial influencers, embedding extremist ideologies into routine gameplay (Schlegel, 2020; Schlegel and Kowert, 2024).

Interviewees highlighted how peer dynamics within gaming platforms further compound risks. For example, when adolescents are surrounded by peers, they experience heightened activation in brain regions associated with social rewards, increasing their susceptibility to peer influence (Chein et al., 2011). This dynamic is exploited in multiplayer games where collaboration is rewarded, even when interactions involve extremist content (Przybylski and Weinstein, 2013). Case studies further illustrate these concerns. In one example, two German children, both aged 12, were exposed to far-right extremist symbolism in the game Roblox and subsequently groomed on Discord. The recruiters manipulated the children’s emotional need for belonging, convincing them to embrace Neo-Nazi ideologies. For example, one child was instructed by the extremist recruiter to perform a Hitler salute at school, demonstrating the profound impact of online radicalization and how it can mobilize action (Koehler et al., 2022). In addition to ideological content, interviewees discussed how adverse offline experiences make children more vulnerable to online radicalization. Factors such as social exclusion, family dysfunction, and psychological distress can increase susceptibility to extremist recruitment (Yuzva Clement et al., 2023; Hutchinson et al., 2025). For instance, adolescents rejected by peers and those experiencing social and cultural isolation are more likely to engage with antisocial media and violent games, which can further exacerbate aggression and radicalization tendencies (Plaisier and Konijn, 2013; Gabbiadini and Riva, 2018). However, exposure to online violent extremist content does not always lead to radicalization or violent action, as individual responses are shaped by a range of factors, including personal resilience, critical thinking skills, social support networks, and preexisting beliefs, which mediate how such content is interpreted and internalized.

While empirical investigations into the mechanisms of radicalization in online gaming environments are still emerging, the dynamics in this study strongly align with established theoretical observations of recruitment and trust-building in online spaces. Digital games are increasingly recognized as influential cultural artifacts in online youth radicalization, owing predominately to the distinctive structural and cultural characteristics and norms they foster (Schlegel and Kowert, 2024, p. 77–79). In contrast to more conventional social media environments, online gaming spaces offer a unique context for cultivating rapid and emotionally charged interpersonal connections, a phenomenon sometimes described as “emotional jumpstarting” (Schlegel and Kowert, 2024, p. 77–79). These shared, intense experiences, such as navigating competitive or stressful in-game situations, can significantly accelerate the development of trust between players (Schlegel and Kowert, 2024). This dynamic is particularly relevant to online youth radicalization, where trust plays a critical role in making individuals more cognitively receptive to extremist views (Kowert, 2014). Within this context, online games are uniquely positioned to facilitate the kind of closeness, or social bonding that precedes ideological indoctrination. These environments not only serve as mediums of communication but also as immersive experiences that can amplify the persuasive power of recruiters through embedded mechanisms of cooperation, competition, and shared identity (Kowert, 2014; Lakhani, 2021; Schlegel and Kowert, 2024).

This dynamic has important implications for how current efforts to mitigate online youth radicalization to violence and approaches to risk and intervention. While policy discussions frequently focus on content moderation and platform accountability, there remains a limited understanding of how immersive digital environments like online games function as sociocognitive environments in their own right. Our cross-sectoral interviews revealed a patchwork of policy awareness: While some stakeholders were attuned to the growing relevance of gaming spaces, many had not yet fully incorporated these environments into risk assessments or intervention strategies. This suggests that current policy and practice may be overlooking not just where recruitment happens, but how it happens, through the slower accrual of trust, the affective resonance of shared play, and the embedding of ideological narratives within these interactions. Moreover, our data underscore the significance of multi-sectoral collaboration, especially when confronting such complex, evolving challenges. Stakeholders recognized the need for more integrated approaches, but commonly cited barriers related to institutional silos, resource constraints, or mismatched priorities. Findings points to a crucial tension: while awareness of online youth radicalization is increasing, the practices and frameworks needed to address it are still developing alongside the technological foundations that are supposedly designed for. Coordination and knowledge-sharing on online gaming technologies will be critical if interventions become proactive and holistic.

Moving forward, policies must evolve beyond punitive or content-centric models and instead embrace a more holistic view of young people’s digital media development. This includes engaging with the relational and cultural dimensions of online gaming, supporting frontline practitioners with clearer guidance, and fostering spaces for young people to build resilience through critical digital literacy and peer supports. Our study contributes to this broader conversation by highlighting the possibilities and current limitations of existing responses, and pointing to the need for proactive, ecologically informed, and context-specific strategies.

### Considerations and limitations

This research does not encompass experiences and perspectives of children or adolescents. Accordingly, the authors have refrained from commenting on the appropriateness or accuracy of the contested concept “online youth radicalization,” purporting to capture experiences and decision-making processes of “at-risk” children and adolescents and characterize the social, cultural, or cognitive-psychological processes that shape their indoctrination. While ethically necessary, excluding direct youth perspectives limits the study’s generalizability, indicating an important direction for future research.

This study does not encompass the knowledge and assessments of non-English-speaking academics, practitioners, and policymakers. Instead, the authors made efforts to engage with a diverse range of English-speaking professionals and encourage future researchers to incorporate multilingual approaches or collaborate with researchers proficient in other languages. Additionally, contextualizing the interviewees’ reflections within national and local policy landscapes is considered essential, and the authors acknowledge the ethical imperative to present findings responsibly across geographic areas.

## Conclusion

This article presented insights drawn from 18 semi-structured interviews with expert researchers, practitioners, and policymakers from universities, P/CVE agencies, and governmental and civil society organizations across the Indo-Pacific, Europe, and North America. A key implication of this research is the necessity for collaboration between counter-extremism practitioners, developmental psychologists, child- and youth-serving organizations, and online gaming industry stakeholders to design interventions that address how youth engages with extremist content and to what effect, youth engage with extremist content at different developmental stages. The unique cognitive and emotional experiences of neurodivergent individuals highlights the need for future research on how specific neurodivergent traits may intersect with extreme gaming adjacent communities. Additionally, gaming architecture has further complicated this threat landscape, providing anonymity, peer validation, and immersive gameplay that can foster their ideological commitment and online socialization.

Accordingly, this study suggests further developing safety-by-design interventions across all relevant digital platforms. Subsequently, rather than primarily focusing on content moderation, prevention strategies prioritizing media and information literacy, digital resilience, critical thinking, ethical game design principles, and the involvement of childcare workers with the appropriate developmental or psychological background are representative of more innovative risk mitigation tactics within online gaming and/or gaming adjacent platforms. This study strongly recommends future initiatives feature interdisciplinary collaborations that are concentrated on understanding the vulnerabilities of child and adolescent development in the current digital age, with particular reference to peer-influence, emotional intensity, and neuroplasticity. These areas call for longitudinal, design-based, and multi-stakeholder research agendas that must be proactive, integrative, and tailored to the distinctive architectures and sub-cultures of online gaming communities. These collaborations must staunchly resist the temptations to over-securitize online gaming communities or the online populations of children and adolescents who are dwelling and developing in these platforms as extremist or terrorist threats.

## Data Availability

Due to the sensitive nature of the material and potential ethical risks to participants, the raw data cannot be made publicly available. The dataset may be provided upon reasonable request and at the discretion of the research team.

## Footnotes

1 Dedicated to our esteemed colleague and dear friend, Mr. Kenton Bell, whose intellectually formidable presence, thoughtfulness, and steadfast commitment to men and masculinities scholarship has been a constant source of inspiration and insight. This article is a tribute to his enduring contributions, which continue to shape and enrich our work and our memories of him.

2 Andrew Tate is a prominent manosphere influencer known for espousing extreme misogynistic views. He is frequently cited by experts as emblematic of harmful gender ideologies affecting young users on gaming and social media platforms, and has been accused of cultivating a “cult-like” influence over his largely male audience through promises of wealth, dominance, and escape from societal constraints.

## In memoriam

Dedicated to our esteemed colleague and dear friend, Mr. Kenton Bell. Whose intellectually formidable presence, thoughtfulness, and steadfast commitment to men and masculinities scholarship has been a constant source of inspiration and insight. This article is a tribute to his enduring contributions, which continue to shape and enrich our work and our memories of him.
